# Endoscopic One-Nostril Transseptal Transsphenoidal Approach for Pituitary Tumors: Back to the Past—A Multi-Center Preliminary Experience and Literature Review

**DOI:** 10.3390/cancers18040592

**Published:** 2026-02-11

**Authors:** Denis Aiudi, Alessio Iacoangeli, Andrea Mattioli, Simone Russo, Massimo Balbi, Stefano Vecchioni, Michele Luzi, Roberto Trignani, Alberto Califano, Ruggero Antonini, Mario Chiapponi, Fabio Greco, Fabrizio Salvinelli, Kenan Arnautovic, Maurizio Iacoangeli, Maurizio Gladi

**Affiliations:** 1 Neurosurgical Department, Marche Polytechnic University, Azienda Ospedaliero Universitaria delle Marche, 60126 Ancona, Italy; s1102391@pm.univpm.it (A.M.); s1118094@pm.univpm.it (A.C.); s11111340@pm.univpm.it (R.A.); s1108096@pm.univpm.it (M.C.); maurizio.iacoangeli@ospedaliriuniti.marche.it (M.I.); maurizio.gladi@ospedaliriuniti.marche.it (M.G.); 2 Division of Neurosurgery, Azienda Ospedaliero Universitaria delle Marche, 60126 Ancona, Italy; stefano.vecchioni@ospedaliriuniti.marche.it (S.V.); michele.luzi@ospedaliriuniti.marche.it (M.L.); roberto.trignani@ospedaliriuniti.marche.it (R.T.); 3 Department of Otolaryngology, Marche Polytechnic University, Azienda Ospedaliero Universitaria delle Marche, 60126 Ancona, Italy; simone.russo@ospedaliriuniti.marche.it (S.R.); massimo.balbi@ospedaliriuniti.marche.it (M.B.); 4 IRCCS INRCA–Istituto di Ricovero e Cura a Carattere Scientifico, Marche Polytechnic University, 60127 Ancona, Italy; 5 Department of Otolaryngology, Fondazione Policlinico Universitario Campus Bio-Medico, Via Alvaro del Portillo, 00128 Roma, Italy; f.greco@unicampus.it (F.G.); f.salvinelli@policlinicocampus.it (F.S.); 6 Semmes Murphey Neurologic & Spine Institute, Memphis, TN 38120, USA; arnautovic@semmes-murphey.com; 7 Department of Neurosurgery, University of Tennessee Health Science Center, Memphis, TN 38163, USA

**Keywords:** pituitary tumor, transseptal, transsphenoidal, endoscopic, mono-nostril

## Abstract

Objective: Endoscopic endonasal transsphenoidal pituitary surgery is a diffuse and well-established surgical technique: over the years, the transseptal approach via a nasal mucosal incision has also gained popularity. Here we describe our preliminary experience with an entirely endoscopic one-nostril transseptal transsphenoidal approach (EONOTTA) for pituitary sellar tumor resection; the surgical corridor runs through the entire length of the nasal septum via an incision in the nasal mucosa. Methods: A total of 40 patients with a midline prevalent pituitary tumor who underwent EONOTTA from January 2022 to June 2023 were retrospectively reviewed for the evaluation of the safety and efficacy of this technique. Results: At 1 year follow-up, all patients had no recurrence, and the degree of tumor resection was comparable to that of the control group undergoing the traditional endoscopic endonasal approach. A low rate of nasal and post-surgical complications occurred; globally, EONOTTA was not time-consuming, and a better functional result was noticed, with a better quality of life for patients. Conclusions: This study confirms, in our preliminary experience, the EONOTTA’s excellent risk–benefit ratio in selected cases; for an experienced multidisciplinary team, it provides a good maneuverability and a functional outcome while preserving the integrity of the nasal mucosa.

## Simple Summary

This study presents the Endoscopic One-Nostril Transseptal Transsphenoidal Approach (EONOTTA) as a minimally invasive surgical technique for selected cases of pituitary macroadenomas. Derived from the classic endoscopic endonasal approach, EONOTTA allows excellent exposure of the sellar region while preserving nasal mucosa and olfactory function. The technique offers comparable disease control to standard endoscopic endonasal approaches, with reduced sinonasal morbidity and postoperative discomfort. Despite limitations such as retrospective design and small sample size, findings support EONOTTA as a safe, efficient alternative for non-extended pituitary tumors requiring limited surgical exposure.

## 1. Introduction

The endoscopic endonasal transsphenoidal surgery is, nowadays, the standard technique used to approach sellar and parasellar tumors, providing better visualization and an improved extent of tumor resection [1,2,3,4]. The two nostrils/four hands technique was introduced to improve the maneuverability of surgical instruments and to increase the accessibility of the anterior skull base, especially in cases of expanded endoscopic endonasal approaches; for this purpose, the nasoseptal flap concept was developed in order to reduce the risk of cerebrospinal fluid (CSF) leakage [5,6]. Since the endoscopic endonasal approach (EEA) is a well-established technique with a low rate of complications, the focus has recently shifted to nasal function preservation; nevertheless, EEA inevitably damages the nasal cavities with possible complications such as hyposmia or synechiae, which are often associated with discomfort and reduced quality of life [7]. To improve sinonasal morbidity, the endoscopic transsphenoidal approach keeps evolving by borrowing the advantages of other and older surgical approaches, such as microscopic transseptal surgery, which reaches the sellar area through the mono-nostril submucosal transseptal corridor [8]. Inspired by this approach, which was once the “gold standard”, several modified endoscopic transseptal or mononostril endoscopic endonasal approaches have been described, and many groups have reported promising functional results. However, these modified techniques, although less traumatic than the classic EEA, still require partial or total bilateral submucoperichondrial and sub-mucoperiosteal dissection or violation of a single nostril via the endoscopic endonasal route. Furthermore, it was questioned whether the benefit of endoscopic endonasal transseptal approaches in preserving nasal mucosa or the mononostril endoscopic endonasal technique could be at the sacrifice of neurosurgical outcomes due to their limited surgical exposure and maneuverability [9,10,11,12,13,14,15]. Therefore, taking inspiration from the various surgical techniques proposed in the literature, the aim of this study is to describe our experience regarding a single nostril transseptal endoscopic approach, performed in selected cases of pituitary macroadenoma, with complete preservation of the nasal mucosa and turbinates. This study presents the results of this technique applied to a cohort of selected patients with an analysis of the safety and the effectiveness on the quality of resection, also considering sinonasal morbidity.

## 2. Materials and Methods

A total of 40 patients, with a midline prevalent pituitary tumor, who underwent EONOTTA from January 2022 to June 2023, were retrospectively reviewed for the evaluation of the safety and efficacy of this technique; both secreting and non-secreting pituitary macroadenomas were included. Neuroimaging inclusion criteria, assessed with MRI-pituitary protocol and CT scan, were midline seated lesions, minimal cavernous sinus invasion (Knosp grade 1–2), or absence of kissing carotids, and exclusion criteria were recurring adenomas previously operated, any kind of previous sinonasal surgery, massive cavernous sinus invasion (Knosp grade 3–4), or the need of expanded endoscopic approaches. Clinical data such as age, sex, smoking habits, possible cocaine abuse, dimension of tumor, any abnormalities in hormonal tests, degree of tumor removal, postoperative complications, and quality of life, including sinonasal morbidity, were collected. Surgical technical details were analyzed, including the type of surgical intervention, duration of surgery, intraoperative complications, intraoperative hematic losses, intraoperative and postoperative cerebrospinal fluid leak rate, and surgical material used for the reconstruction; the overall presence of post-operative endocrinological complications (e.g., diabetes insipidus) was also tracked. A head CT (GE HealthCare, Chicago, IL, USA) scan was always performed on postoperative Day 1 to detect any intracranial bleeding or pneumencephalus, together with an MRI (GE HealthCare, Chicago, IL, USA) study before discharge to verify the degree of tumor removal; an endoscopic check-up and endocrinological consultation were performed as well. All patients underwent a follow-up at 1, 3, 6, and 12 months, also evaluating the quality of life in relation to any sino-nasal disorders.

To ensure a meaningful comparison, a control group was also included, consisting of 40 patients who underwent the standard binostril endoscopic endonasal approach (EEA) for pituitary adenoma resection during the same study period. These patients were selected retrospectively from the same institutional databases and operated on by the same surgical teams under identical perioperative protocols. The inclusion and exclusion criteria applied to the control group were identical to those used for the EONOTTA cohort, namely pituitary macroadenomas located predominantly in the midline, minimal or absent cavernous sinus invasion (Knosp grade 0–2), and absence of previous pituitary or sinonasal surgery. Patients with recurrent lesions, extensive parasellar or lateral tumor extension, or the need for extended transpterygoid approaches were excluded. This selection ensured that both cohorts were comparable in terms of tumor size, Knosp grade, functional status, and surgical eligibility. The binostril EEA control group was treated using the standard two-nostril, four-hand technique, involving partial removal of the posterior nasal septum to create a common working corridor. The same endoscopic equipment, magnification, and reconstruction materials were used in both groups. Postoperative management and follow-up evaluations were performed according to the same institutional protocol as for the EONOTTA cases. For the statistical analysis, continuous variables such as age, tumor size, and hospital stay were expressed as mean ± standard deviation (SD) and compared between groups using the Student’s *t*-test or Mann–Whitney U test as appropriate. Categorical variables, including sex, Knosp grade, tumor type (secreting vs. non-secreting), gross total resection rate, and postoperative complications, were analyzed using the chi-squared test or Fisher’s exact test. Statistical significance was defined as *p* < 0.05. Statistical analyses were performed using MedCalc Statistics software (version 23.2.0). Given the retrospective design of the study, which involved only the analysis of fully anonymized data from patients who underwent standard surgical procedures as part of routine clinical care, with no additional interventions or risk for the patients, formal approval by an ethics committee was not required, in accordance with applicable institutional and national regulations. The study was conducted in compliance with the principles of the Declaration of Helsinki.

## 3. Results

A total of 80 patients with a midline prevalent sellar tumor who underwent endoscopic transsphenoidal pituitary surgery from January 2022 to June 2023 were reviewed for evaluation of the safety and efficacy of the approach. Forty patients were treated using the EONOTTA, while forty were treated during the same period using the standard binostril EEA, selected as the control group based on comparable radiological and clinical characteristics.

The two groups were homogeneous in terms of baseline demographic and tumor-related variables, including patient age, sex distribution, tumor diameter, proportion of functioning adenomas, Knosp classification, and surgical eligibility. No statistically significant differences were observed between the cohorts in these parameters. The rates of gross total resection (GTR) and hormonal remission were also similar between the two surgical techniques. The data for the main variables in both groups are summarized in Table 1.

At follow-up after 1 year, no recurrences, no CSF leaks, and no nasal mucosa scarring related to the approach were registered. The degree of tumor resection was comparable to the control group undergoing the traditional endoscopic endonasal approach, and a low rate of nasal complications occurred: 1 septal perforation and 1 mucosal dehiscence.

The low rate of postoperative morbidity was also confirmed by a single case of post -procedural synechia. Although a longer operative time was observed in the initial cases, overall, the EONOTTA did not prove to be time-consuming, and a superior cosmetic outcome was consistently noted at follow-up. Clinical and laboratory results at 12 months are summarized in Table 2.

The mean operative time in these series was 150.4 min and the mean length of stay in the hospital was 4.2 days. Gross total resection (GTR) was achieved in 85% patients. Hormonal remission, as confirmed by serial postoperative serum hormone assessments, was achieved in 75% of patients at the last follow-up. Regarding surgical complications, intraoperative cerebrospinal fluid (CSF) leakage occurred in 17.5% of cases. Other postoperative complications included diabetes insipidus in 17.5% of cases and anterior pituitary insufficiency requiring hormone replacement therapy in 27.5%. No ICA injury or death happened in this series. More than half of the patients (52.3%, 67/128) suffered from decreased visual acuity and visual field defects before surgery. Most of the patients (82.1%, 55/67), more than half of the patients in both groups (21/40 in group 1, 23/40 in group 2), suffered from decreased visual acuity and visual field defects before surgery. Most of the patients (80.9% in group one, 78.2% in group 2) displayed improved visual status and no patient presented a worsened visual status.

At two weeks postoperatively, the most frequent sinonasal complaints included headache (15.6%), nasal discharge (11.7%), nasal obstruction or breathing difficulty (10.2%), hyposmia (7.8%), nasal synechiae (2.3%), and epistaxis (0.8%). By one month after surgery, the majority of symptoms had resolved, with only a small proportion of patients reporting residual nasal discharge (4.7%), as well as hyposmia (3.9%), breathing difficulty (3.1%), and headache (2.3%). At the three-month follow-up, almost all sinonasal symptoms had completely resolved. These findings are summarized in Table 3.

Regarding sinonasal quality of life, as assessed by the ASK Nasal-12 questionnaire, scores showed a transient postoperative increase at two weeks, followed by a significant reduction at one month after surgery. By three months postoperatively, ASK Nasal-12 scores had returned to baseline values. Consistently, evaluation using the Lund–Kennedy endoscopic score demonstrated that, at one month, the nasal cavity of patients undergoing the EOTA approach had recovered to baseline conditions. Similarly, the odor identification test revealed a restoration of olfactory performance comparable to preoperative baseline levels one month after surgery [16,17,18,19,20,21].

The study showed that, compared to the control group, where the surgical approach was a traditional endoscopic endonasal one, the operating times were progressively reduced with the surgeon’s increasing experience in performing the EONOTTA, reaching a comparable gross total resection value, with less destruction of the nasal mucosa and a lower incidence of complications such as cerebrospinal fluid fistula and anatomical-functional nasal-sinus preservation, as evidenced by endoscopic and follow-up checks.

### Clinical Case

**Figure 1:**
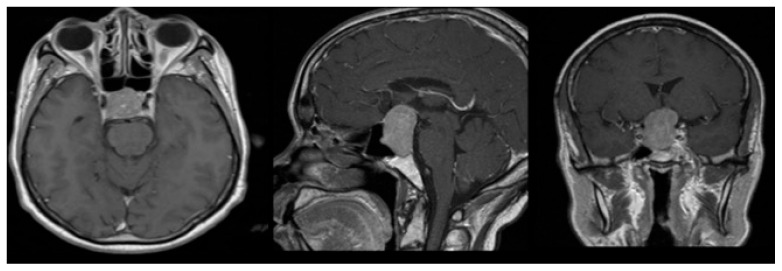
Axial, sagittal, and coronal MRI performed before surgery.

A 58-year-old woman presented with severe hyponatremia, headache, and bitemporal hemianopsia. As shown in Figure 1, preoperative MRI revealed midline pituitary macroadenoma extending suprasellarly, compressing the optic chiasm and not invading cavernous sinuses.

**Figure 2:**
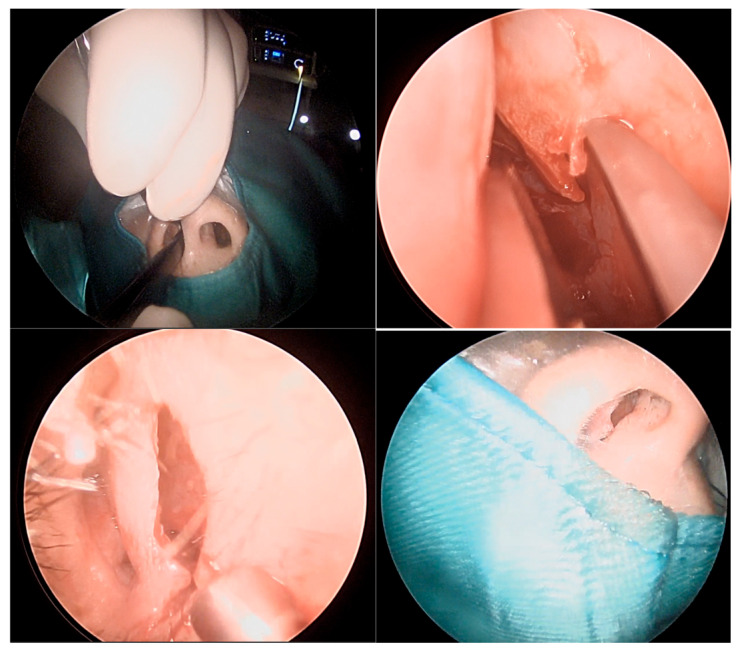
Mono-nostril approach and septal mucosal incision.

The patient underwent an Endoscopic One-Nostril Transseptal Transsphenoidal Approach (EONOTTA) for tumor removal. Figure 2 shows the right mono-nostril approach and the septal mucosal incision with the initial submucosal dissection along the septum.

**Figure 3:**
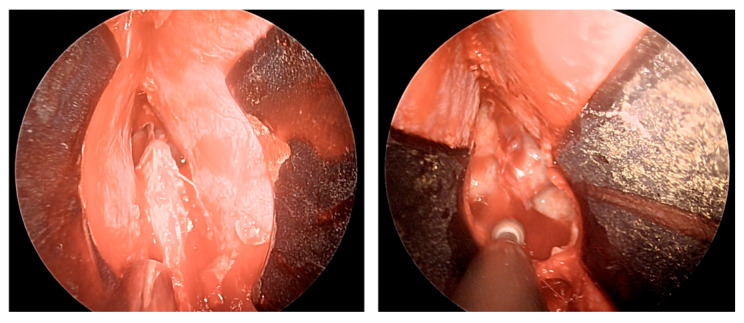
Positioning of self-retaining nasal speculum between nasal septum and mucosal flap and drilling of the sphenoid bone.

Once the mucosal dissection is completed, a self-retaining nasal speculum is placed between the mucosal flap and the cartilaginous nasal septum, obtaining sphenoidal exposure (Figure 3).

**Figure 4:**
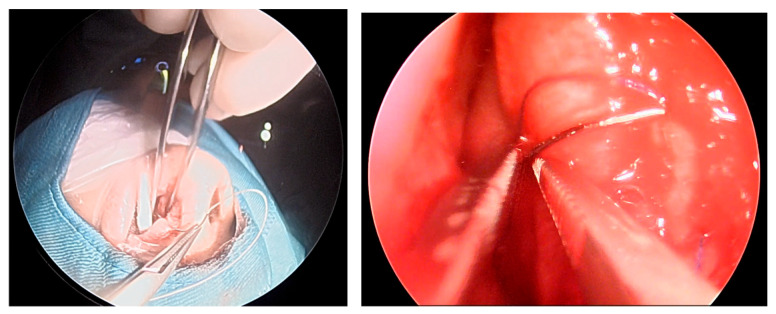
Placement of simple interrupted suture.

After removal of the adenoma and placement of autologous adipose tissue, as shown in Figure 4, unilateral mucosal reconstruction is performed by placing a simple interrupted suture through the mucosal flap and cartilaginous septum.

**Figure 5:**
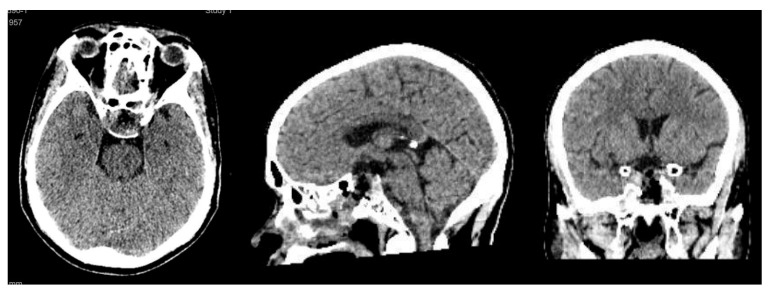
Axial, sagittal, and coronal CT scan performed on postoperative Day 1 after surgery.

Postoperative imaging demonstrated near-total resection of the lesion with decompression of the optic apparatus and restoration of normal sellar anatomy (Figure 5).

**Figure 6:**
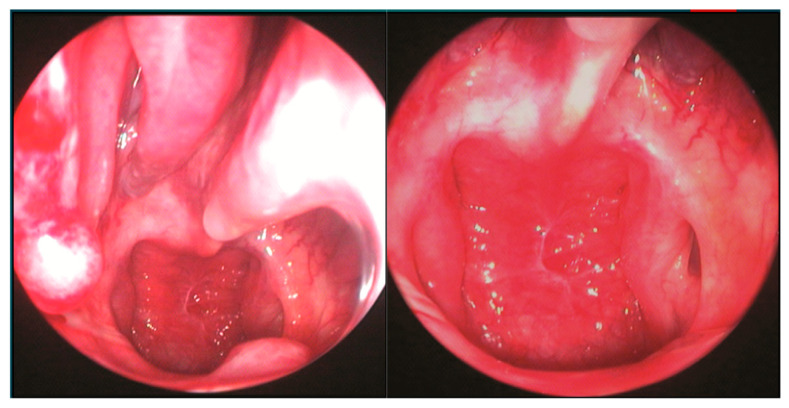
ENT 4 days after surgery and 30 days later.

The patient underwent endoscopic ENT follow-up 4 days after the operation and 30 days later (Figure 6).

**Figure 7:**
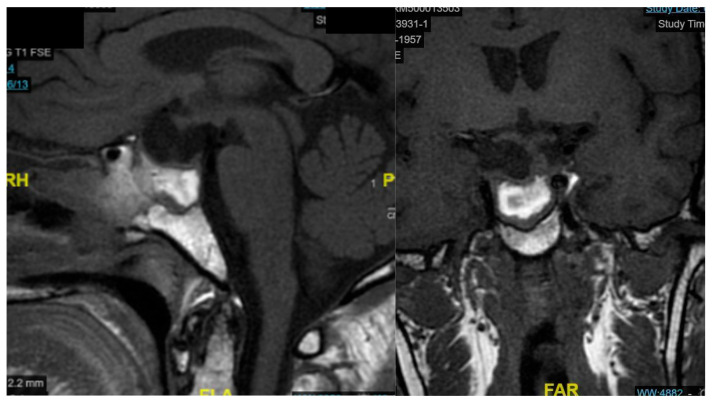
Sagittal and coronal MRI one month after surgery.

The patient showed significant clinical improvement, with resolution of visual symptoms and normalization of serum sodium levels. No postoperative cerebrospinal fluid leakage, nasal crusting, or olfactory dysfunction was observed. The MRI examination performed one month later shows packing material, with no radiological evidence of residual lesion (Figure 7).

The EONOTTA proved effective in achieving optimal tumor control and functional recovery while minimizing sinonasal morbidity. This case highlights the value of the transseptal endoscopic technique in selected pituitary macroadenomas without major lateral extension, ensuring both surgical efficacy and improved postoperative quality of life.

## 4. Discussion

Pituitary adenomas are benign clonal neuroendocrine neoplasms arising from adenohypophyseal cells and are currently classified under the broader entity of pituitary neuroendocrine tumors (PitNETs) [22]. The surgical management of pituitary lesions has evolved substantially over the past century. Early descriptions of pituitary surgery via a sublabial transseptal route were provided by Halstead in 1910 and later refined by Harvey Cushing. A major technological advancement occurred in 1961, when Gérard Guiot incorporated the operating microscope and introduced endoscopic assistance to improve visualization of tumor resection, although its application was constrained by the limited optical performance of early endoscopes. The modern era of endoscopic endonasal surgery began in 1992, when Roger Jankowski reported the first fully endoscopic endonasal procedure, a technique that has since become the foundation for most contemporary endoscopic approaches to the sellar region [3,23,24]. In the literature, many endoscopic techniques for approaching sellar lesions are reported, but they can, overall, be summarized in two categories: the classic EEA performed in both or single nostrils or transseptal techniques that involve both sides of the septal mucosa. In this article, we would like to propose the endoscopic one-nostril transseptal transsphenoidal approach, which we have applied in selected cases of pituitary macroadenoma and have observed to be effective in controlling the disease with minimal invasiveness for the sinonasal structures. The introduction of this technique into our clinical practice, which is nothing more than a revisitation of the old microscopic transseptal approach, arises from the idea that, in selected midline cases, when there is no need to use an extended endoscopic approach, a unilateral transseptal surgical corridor is sufficient to gain a good exposure of the disease and a comfortable working angle. It allows two surgeons to work together at the same time, thus addressing the removal of the tumor as in the classic EEA, with less morbidity affecting the nasal structures and often without the need for pedunculated flaps, as repositioning the vascularized mucosa of the septum unilaterally acts as a flap itself [25,26].

The endoscopic one-nostril transseptal transsphenoidal approach has been increasingly compared with more conventional binostril endoscopic and microscopic techniques, demonstrating a specific balance of advantages and limitations. Mononostril approaches tend to preserve a greater amount of nasal mucosa and olfactory epithelium, which may reduce early sinonasal morbidity—such as crusting, epistaxis, and olfactory disturbances—although long-term sinonasal quality-of-life outcomes generally remain comparable across endoscopic techniques [27]. Operative duration is often similar or marginally shorter than in binostril approaches in experienced hands, largely due to reduced intranasal dissection. Blood loss is frequently lower than in classic microscopic transseptal procedures, benefiting from improved visualization and targeted hemostasis [28]. Surgical outcomes, including gross total resection rates and endocrinological results, remain comparable between approaches, although the binostril EEA offers greater instrument maneuverability for lesions with significant lateral extension [29]. Reconstruction strategies also differ substantially: the binostril EEA facilitates the harvesting of a pedicled nasoseptal flap for large skull-base defects, whereas the one-nostril transseptal approach typically employs direct re-approximation of the unilateral mucoperichondrial flap. This reduces donor-site morbidity but limits availability of vascularized flaps for extensive defects [13]. Overall, current evidence suggests that the one-nostril transseptal approach provides a minimally invasive alternative with reduced nasal morbidity and comparable efficacy in appropriately selected cases.

**Figure 8:**
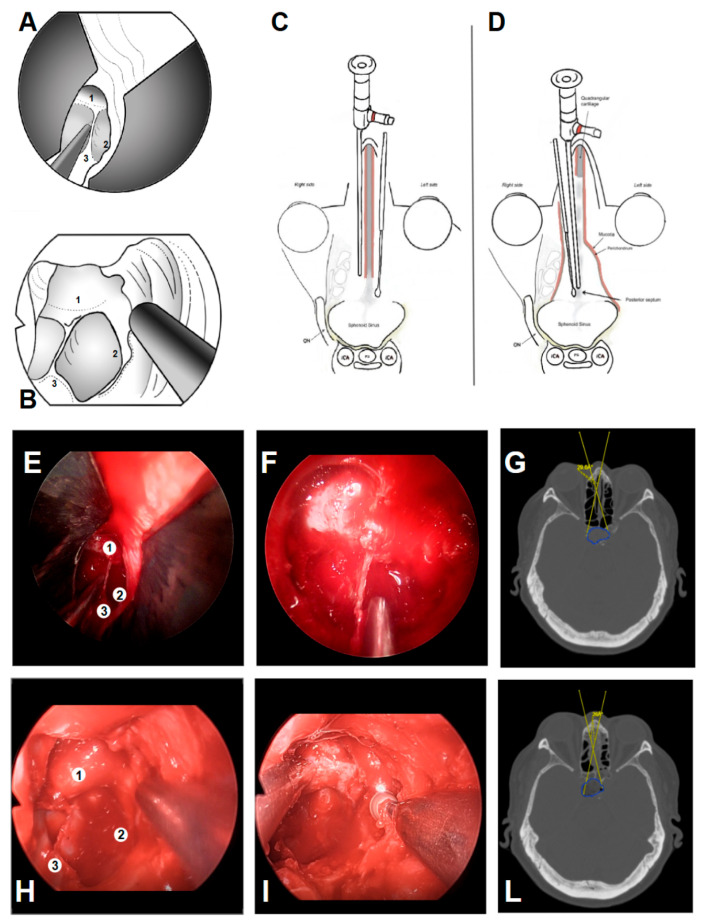
Comparison of the EONOTTA (**A**) and binostril EEA (**B**). EONOTTA (**E**,**F**) and binostril EEA (**H**,**I**) initial and deep endoscopic views showing the initially narrow entry corridor, with similar deep visualization of landmarks such as the sellar bulge (1), posterior sphenoid sinus wall (2), and septum (3). (**C**,**D**) Pre-operative CT scans demonstrating the expected working angles and the corresponding tumor regions for the EONOTTA patient ((**G**): 29.6°) and the binostril EEA ((**L**): 29.7°).

In our experience, even the initially seemingly narrow mononostril corridor still allows adequate deep exposure of the sphenoid and parasellar landmarks, comparable to the classic binostril EEA [24,30,31] (Figure 8).

In some cases of large CSF fistulas, the classic pedunculated nasoseptal flap may still be necessary for skull base reconstruction but harvesting of this vascularized flap involves cutting the mucosa and is associated with an increased risk of postoperative nasal crusting, raising concern about worsening postoperative sinonasal quality of life. The EONOTTA offers several anatomical and functional advantages from a sinonasal perspective, as it follows a strictly midline corridor and is therefore not influenced by anatomical variations such as turbinate hypertrophy or septal deviation. Dissection is performed within an avascular subperichondrial and subperiosteal plane, which is typically associated with minimal intraoperative bleeding, thereby reducing the risk of injury to the posterior nasal artery. Moreover, preservation of the middle and superior turbinates may theoretically lower the incidence of postoperative epistaxis and olfactory dysfunction. An additional advantage of this approach is the feasibility of harvesting a nasoseptal flap during the same procedure or preserving it for potential use in revision surgery, when required [10,16,26,32].

Functional complications related to the transseptal approach are rare and endonasal anatomy is globally preserved in most cases. Although initially more time-consuming than classic EEA, the transseptal approach is nevertheless familiar for ENT surgeons due to the similarities with conventional septoplasty techniques. Endoscopic transseptal technique was introduced therefore with the aim of reducing sinonasal morbidity and improving sinonasal quality of life, but the speculum restricts the bimanual handling of instruments, and therefore it would be suboptimal in cases of tumors with significant lateral extension and in all those patients who require an extended endoscopic approach [16,33,34]. Our study revealed that the patients who underwent EONOTTA had less postoperative pain, better nasal outcomes with intact olfactory function than the standard endoscopic transnasal approaches, and a degree of disease control comparable to the latter surgical technique. Based on our preliminary experience, the EONOTTA represents a valuable surgical option that, in carefully selected cases, integrates the benefits of both endoscopic and microscopic techniques. Specifically, it offers neurosurgical outcomes comparable to those achieved with standard endoscopic transnasal approaches, while simultaneously minimizing sinonasal morbidity, a feature traditionally associated with microscopic transseptal surgery. Nevertheless, despite its appeal, this transseptal endoscopic technique is not without limitations. Elevation of the septal mucosal flap may be technically challenging in patients with a history of prior nasal procedures, such as septoplasty or rhinoplasty, and lesions exhibiting significant lateral extension may not be amenable to complete resection through this corridor [5,16,28,29]. Furthermore, it is very difficult in our experience to try to define and quantify the relative contribution of postoperative nasal symptoms to overall quality of life. Obviously, the perception of nasal disturbance is difficult to ascertain because it impacts the quality of life differently, also depending on the expectations and what the patient deals with within his working life. This transseptal surgical technique clearly benefits from a multidisciplinary team composed of a neurosurgeon and an ENT surgeon; the latter is essential to perform the rhinologic phases, avoiding mucosal lesion or septal perforation during the first stages of the approach, and to prevent time consumption.

Several limitations have been encountered in this work. Firstly, the study was retrospective in nature and included a small-size sample; results still require validation with randomized-controlled studies. Histologically, only pituitary adenomas were treated with the transseptal approach; it remains unclear whether these findings can be generalized to other sellar pathologies treated with endoscopic endonasal techniques. In addition, the current analysis is restricted to a follow-up duration of one year after surgery. As a result, the true incidence of late-onset complications, particularly septal-related morbidity, may not be fully captured, underscoring the need for longer-term follow-up to more accurately assess these outcomes [7,16,31,33,35].

The EONOTTA is, in our opinion, a safe and minimally invasive approach to address pituitary macroadenomas without major lateral extension that enables excellent exposure of the intrasphenoidal anatomical landmarks with maximal preservation of the nasal mucosa and better nasal recovery than classic endoscopic endonasal approaches. This approach enables intrasellar working, without major drawbacks, with a two-hand to three-hand technique. It might be considered as an alternative surgical technique for addressing pituitary adenoma under selected circumstances. The results of this study confirm the safety and the efficiency of this approach for non-extended pituitary surgery.

## 5. Conclusions

According to our experience, this study confirms the excellent risk–benefit ratio supporting the use of the transsphenoidal endoscopic transseptal approach in selected cases; this working corridor for pituitary surgery was found to be easy for an experienced multidisciplinary team to use, providing good maneuverability and an effective approach with a minimal rate of post-operative complications.

## Figures and Tables

**Table 1:** Baseline demographic, tumor, and surgical outcome characteristics of patients undergoing the EONOTTA and the standard binostril EEA.

Variables	Group 1 (*n* = 40)	Group 2 (*n* = 40)
Mean Age	50.8	51.4
Sex (M/F)	17/23	19/21
Tumor diameter (mm)	24.8	25.3
Functioning Adenoma %	45%	40%
Knosp grade	Knosp 1 *n* = 24 Knosp 2 *n* = 16	Knosp 1 *n* = 22 Knosp 2 *n* = 18
Pre-op visual deficit	21/40	23/40
Gross Total Resection %	85.0%	87.3%
Operative time (min)	150.4	161.1
Hospital stay (days)	4.2	4.3
Hormonal Remission %	74.7%	76.8%

**Table 2:** The 12-month postoperative results.

	Group 1	Group 2
CSF leakage (%)	17.5	15.0
Visual improvement (%)	80.9	78.2
Post-op Diabetes Insipidus (%)	17.5	20.0
Pituitary insufficiency (%)	27.5	30.0
No Hormonal Variation (%)	45.0	40.0
Cortisol Variation (%)	22.5	20.0
Thyroid hormones variation (%)	17.5	15.0
Gonadal hormones variation (%)	10.0	12.5
Multiple hormonal variation (%)	5.0	7.5

**Table 3:** Reference cohort’s main sinonasal complaints.

Group	Symptom	2 Weeks	1 Month	3 Months
1	Headache	15.6%	2.3%	~0%
	Nasal discharge	11.7%	4.7%	~0%
	Trouble breathing	10.2%	3.1%	~0%
	Hyposmia	7.8%	3.9%	~0%
	Nasal synechia	2.3%	0%	0%
	Epistaxis	0.8%	0%	0%
2	Headache	19.5%	6.3%	~0%
	Nasal discharge	14.2%	6.6%	~0%
	Trouble breathing	12.5%	5.1%	2%
	Hyposmia	11.9%	6.9%	5%
	Nasal synechia	3.6%	0.5%	0%
	Epistaxis	2.5%	0%	0%

## Data Availability

The original data presented in this study are available on reasonable request from the corresponding authors. The data are not publicly available due to privacy concerns.
